# Learning from Ebola Virus: How to Prevent Future Epidemics

**DOI:** 10.3390/v7072797

**Published:** 2015-07-09

**Authors:** Alexander S. Kekulé

**Affiliations:** Institute for Biosecurity Research, 06180 Halle, Germany; E-Mail: kekule@ibs-halle.de; Tel.: +49-345-1710-6633

**Keywords:** Ebola, epidemic, infectious diseases, WHO, International Health Regulations

## Abstract

The recent Ebola virus disease (EVD) epidemic in Guinea, Liberia and Sierra Leone demonstrated that the World Health Organization (WHO) is incapable to control outbreaks of infectious diseases in less developed regions of the world. This essay analyses the causes for the failure of the international response and proposes four measures to improve resilience, early detection and response to future outbreaks of infectious diseases.

## 1. Introduction

Ebola virus has been raging through Western Africa for seventeen months by now. The number of new cases is declining but an end to the epidemic is not in sight. The World Health Organization registered about 27,500 cases thus far, of which 11,220 have died. It will probably take several more months until the largest EVD outbreak in history can be declared over. Guinea, Liberia, and Sierra Leone will suffer from the economic consequences for many years to come. Scientists and politicians now unanimously argue that international aid came too late and was for the most part ineffective. The WHO in particular has been facing intense criticism not having reacted appropriately to the outbreak. The horrendous images coming out of Western Africa and concern for their own safety have finally woken up the industrialized nations.

How, then, can we protect ourselves against future outbreaks of deadly microbial diseases?

## 2. Causes for the Catastrophe

On 26 December 2013, an 18-month-old boy named Émile fell ill with high fever and severe diarrhea in a village of the Guéckédou Prefecture in the south of Guinea. Today, we suspect that he might have been infected by a fruit bat or some other wild animal which carried the virus into that area. By the time Émile died, his sister, his mother and his grandmother had already been infected. As more and more people fell ill, the local health authorities suspected cholera or Lassa fever to be the cause, both of which are common in that region.

On 13 March 2014, the Guinean Ministry of Health called WHO and Doctors Without Borders (*Médecins sans Frontières*, MSF) for support. Eight days later, the message arrived from a special laboratory in Lyon (France): it is Ebola and, moreover, it is the most deadly strain “Zaire” [sic].

At this point in time, MSF had already begun to isolate patients and to trace contacts in Guéckédou. The reports had often mentioned “hiccups”—a clinical sign that does not fit cholera or Lassa fever, but is frequently observed with Ebola virus disease (EVD). In an EVD outbreak it is essential to isolate all patients and their contacts as early as possible, and MSF immediately sent a specialized team into the affected region.

At WHO in Geneva, however, the reports from Western Africa were received in a markedly more relaxed manner. The Director-General, Dr. Margaret Chan had, in the past, been accused of alarmism. During the “swine flu” pandemic, she had declared the highest alert level, in spite of the relatively harmless manifestations of the disease. Then, during the years that followed, Dr. Chan dissolved WHO’s epidemic and pandemic response unit and split its duties among other departments. This move fit into the strategic turnover that Dr. Chan had pursued since she had taken up office in 2006: WHO should focus on technical advice, and leave practical disease control with the member states.

Until the middle of May 2014 the number of new Ebola virus infections remained stable. During this eclipse of the epidemic, only nine patients were treated in Guinea. Liberia, alerted by the outbreak in the neighboring country, had successfully isolated about a dozen cases. There were no more reports of new cases for several weeks in these two countries.

Sierra Leone had engaged the US firm Metabiota from the start of the epidemic to search for EVD cases in the border regions to Guinea. However, the professional virus hunters did not find any trace of Ebola virus in over 160 samples. On the basis of this information, President Ernest Koroma declared his country “Ebola-free” up until the last week of May 2014. WHO adopted this assertion unchecked in its reports. This would later prove to be the most fatal mistake in the initial stages of the epidemic.

Today it is clear that Ebola virus had already spread in Sierra Leone very early in the epidemic. MSF was told during their investigations in March 2014 of suspected EVD cases beyond the borders in Sierra Leone and immediately issued warnings to the Ministry of Health and WHO office in Freetown. There, the preferred reaction was to believe the reassuring information of the American consulting firm. Up until 24 May, WHO described the situation in Liberia and Sierra Leone as “stable”.

Then, after three weeks of deceptive calm, the viral storm broke loose. On 25 May, Freetown confirmed the first EVD case and, within a few weeks, hundreds more followed. The hidden outbreak in Sierra Leone had sparked a smoldering wildfire in Guinea and Liberia. Because of the high number of unknown cases, the critical mass, which a virus needs for explosive dissemination, had been reached. At least 4600 people died between June and October 2014 alone.

Facing this dramatic situation, the WHO Director-General decided to wait another two months before she declared the EVD outbreak a Public Health Emergency of International Concern (PHEIC). The legal definition of the PHEIC had already been fulfilled in March 2014: an extraordinary event must constitute a public health risk to other states through the spread of disease and potentially require coordinated international response.

The tasks of WHO and its 194 member states in the face of an epidemic are laid down in the International Health Regulations (IHR) of 2005. After declaring a PHEIC, the Director-General appoints a specialized Emergency Committee and issues “temporary recommendations” as to how the epidemic should be tackled. If necessary, WHO has the right to check information provided by affected countries regarding the nature and the spread of an infectious agent through its own investigations. Although the recommendations are not legally binding, the declaration of a PHEIC exerts considerable political pressure both on the affected countries to cooperate and on other states to provide financial aid. Therefore, according to the original intention of the IHR, WHO should take on the coordination of the fight against the epidemic.

The no-longer-disputed fact that WHO reacted too late was based on a fatal misjudgment of the initial epidemic in Western Africa. EVD is, next to rabies, one of the infectious diseases with the highest lethalities. On the other hand, Ebola virus is only transmitted by close contact and those seriously ill with the disease can hardly move. For this reason, earlier EVD epidemics, which occurred in the remote villages of Equatorial Africa, had always burnt out after two to three months.

Dr. Chan and the WHO representatives of the three affected countries had good reason to hope that the Western African epidemic would pass quickly too. The governments of Conakry, Monrovia, and Freetown for their part had no interest in being burdened with travel and trade restrictions. Hence they delivered spuriously low case numbers to Geneva for months and declared the situation as being under control. The common interest of local governments and WHO representatives to play down the problem resulted in fatal negligence, for which the affected countries, and the rest of the world, had to pay a high price in the following months.

On 25 July, Nigeria confirmed its first EVD case. One week later, two American aid workers, who had become infected with the virus in Monrovia, were flown out to the USA. On 8 August 2014 Dr. Chan finally declared the EVD epidemic a Public Health Emergency of International Concern.

By that time very few still believed that WHO, after months of procrastination and misjudgments, could lead an international emergency deployment in Western Africa. UN Secretary General Ban Ki-moon told the WHO leader that he was going to appoint a new UN mission to coordinate the Ebola response. On September 19, the UN Mission for Ebola Emergency Response (UNMEER) was initiated. It is the first health mission outside of WHO in the history of the United Nations, giving a clear signal that the sole UN agency for health could not be relied on to manage a multi-national outbreak.

At the beginning of September, Ellen Johnson Sirleaf, President of Liberia and respected Nobel Peace Prize winner, wrote a personal letter to the leaders of Australia, Brazil, China, Germany, India, Japan, Cuba, Russia, South Africa, and the USA. Shortly after that, President Barack Obama announced the greatest humanitarian aid program in history: 3000 soldiers were to construct treatment centers in Liberia for a total of 1700 EVD patients. China and Germany also pledged to build treatment centers in Monrovia.

However, progress in international response was sluggish. By August, the few operational treatment centers in Western Africa had been overwhelmed by patients. At the same time, officials in Washington, Paris, and Berlin discussed protective equipment, disinfectants, tent constructions, liability issues and medevac procedures for potentially infected aid workers.

In Berlin, an interagency working group led by the chancellery dealt with the many technical, logistical and legal questions (The suggestion made by the author of this article (A. Kekulé, Report for the German Federal Foreign Office, 17 September 2014) to immediately train German aid workers with MSF in Brussels and Western Africa and to construct treatment centers in tents according to the MSF concept, was dismissed as impractical at that time). In recognition of an eminent lack of resources, the federal government finally commissioned the German Red Cross to construct and run an Ebola treatment center in Monrovia, with technical support provided by the German Army (*Bundeswehr*). In contrast to the rather improvised MSF camps, the made-in-Germany treatment center was to be equipped with proper electricity and water supply systems, solid walls, stormproof roofs and concrete foundations. On 23 December 2014, the facility was eventually finished. The necessary personnel, who received a perfect preparation in a purpose-built German training center, were supposed to follow until mid-January. At this point, the MSF 250 bed facility in Monrovia was half-empty because the epidemic had been declining since October. Furthermore, the USA and China had created their own treatment centers with several hundred beds. As a consequence, not a single EVD patient has ever been treated in the German facility.

## 3. Lessons Learned

From the mistakes and the limited successes of the response to the EVD epidemic in Western Africa, we are able to gain five essential insights:

### 3.1. Naturally Occurring Outbreaks do not Happen Suddenly

The West African EVD epidemic started long before little Émile became infected. Current genetic research suggests that Ebola virus had been present in the Western African rainforest for more than ten years before the current present outbreak (Scientists from the Bernhard Nocht Institute in Hamburg found signs of Ebola virus infections in the East of Sierra Leone as early as 1982. However, because detection methods were not reliable in those days, these findings were not investigated further). The migration of Equatorial African fruit bats, which are regarded as a possible source of the epidemic, had been observed for some time. With better preparation and sensitive epidemic surveillance in place, Western Africa could have been warned about Ebola virus.

### 3.2. The Fight against Epidemics Starts with Humans

In the remote regions of the Equatorial African rainforest, where Ebola virus has been present for a long time, people with uncommon fevers are treated by an old woman in a hut outside the village, who everyone avoids during that time: without scientific knowledge, people have developed an appropriate method of isolation (From a virologist’s point of view this makes perfect sense, because older people are more likely to have contracted and survived EVD, and could therefore have become immune). In Western Africa, the people have learned how to protect themselves against EVD-like illnesses: avoid any contact with the sick and the dead, as well as with strangers. Tens of thousands of local community health workers carried this message to the most remote villages. This simple rule of behavior is the main reason why new infections have dropped since October 2014.

The statisticians of the US Centers for Disease Control and Prevention (CDC) and WHO did not take this learning process into account when they predicted at the end of September 2014 that in Liberia and Sierra Leone, alone, the number of EVD cases would rise up to 1.4 million by mid-January 2015 (The CDC estimated 550,000 recorded cases and a factor of 2.5 for underreporting. Extrapolating the calculations of WHO, which published a prognosis only until November 2014, up to 500,000 cases were to be expected by end of February 2015). (The author of this article vehemently disputed those figures at that time. Considering that the affected people would autonomously change their behavior, he predicted that the case numbers would start to drop from November 2014 onwards and that by January 2015 the epidemic would largely be under control (A. Kekulé, Report for the Commission on Civil Protection at the German Federal Ministry of the Interior, 13 October 2014)). On the basis of these pessimistic forecasts, many countries, including Germany, devised long-term aid programs, with the result that most of the treatment centers were only completed when the epidemic had already abated.

Knowledge of culture and living conditions of the affected populations is essential in the fight against epidemics. The fact that WHO, as part of its restructuring policies, decided to let go of almost all its anthropologists, was a grave mistake.

### 3.3. Treatment Centers are the Most Effective Form of Emergency Aid

The behavioral learning process takes time, especially where religious and ethnic customs are involved. During the initial phase of an epidemic, the pathogenicity of the organism determines how quickly it spreads. In highly contagious virus diseases, every patient infects a large number of other individuals. The average number of persons infected by one patient is the *basic reproduction number,* *R_0_* (“R-naught”). For measles, one of the most contagious diseases, *R_0_* is approximately 15. EVD, in contrast, is only transmitted through contact with bodily fluids. At the beginning of the epidemic, when the population did not yet know how to protect itself, *R_0_* had a value of about 2. In order to stop the spread of a disease, the value of *R_0_* must be brought below 1. As long as no vaccine is available, there is only one way to accomplish this task: the infected must be isolated immediately and efficiently.

For this purpose, mobile Ebola Treatment Centers (ETCs) have proved their usefulness during earlier outbreaks. The current epidemic showed that isolation at home (which would be an alternative in highly developed countries) is not feasible under basic living conditions. In addition, it has become clear that simple infusions, to replenish the water and salt losses from fever and diarrhea, can save many patients.

For the isolate-and-treat strategy to work, sufficient capacity must be constructed very quickly. During the most intensive phase of the Western African epidemic, the ETCs were so overcrowded that they could hardly administer infusions. The patients understood quickly that they would only be isolated, but not treated. Understandably, many EVD patients preferred to stay, and to die, at home. Thousands of infected hid away from the aid workers, some even fled from the treatment centers.

### 3.4. In an Emergency, Only Things Will Work That Have Worked Already Before

*Médecins sans Frontières* had more than ten years of experience with outbreaks of EVD and similar diseases. Therefore, it was no coincidence that a MSF specialist thought of Ebola early, having heard only of some clinical symptoms, and reacted appropriately. The plans and the packing lists for ETCs were in the drawer and experienced personnel were available (although not in sufficient numbers). The International Federation of Red Cross and Red Crescent Societies (IFRC) was also able to utilize its earlier experience with EVD and opened its first ETC on 23 September in Sierra Leone, which was still early enough to help hundreds of patients.

The mobile field laboratories, which were provided mainly by the EU and the USA, proved to be successful. The first unit of the European Mobile Laboratory (EMLab) program, managed by the Bernhard Nocht Institute in Hamburg, started operations in Guéckédou in the first days of April 2014.

In contrast, the aid organization Samaritan’s Purse, which had no experience with Ebola outbreaks, had to close its ETC in Liberia after two aid workers became infected. The effort led by the United States was similarly ill-planned: 2800 soldiers built 17 ETCs in Liberia, but there was no medical staff to run the centers. The only US facility that ever came into operation is a small, high-tech hospital in Monrovia, designed exclusively for the treatment of aid workers.

### 3.5. Epidemic Control Must Be Fast and Flexible

Epidemics jump from one location to another at lightning speed and countermeasures must be able to follow. MSF’s camp-type treatment units and the mobile field laboratories were successful because they could be moved quickly and, if necessary, expanded in a modular way. The UK, like all other governmental bodies, could only put its aid program into practice very late. They invested in mobile tent construction, similar to those of MSF, which were then handed over to the management of non-governmental aid organizations. From 5 November until 15 December, at a time when the epidemic was still at its height in Sierra Leone six successful UK treatment centers went operational in that country.

## 4. Four Things that Should Be Done Now

On the basis of the experiences in Western Africa, four important measures for the protection against future epidemics are to be recommended:

### 4.1. Integration of Epidemic Preparedness into International Development Policy

Ebola virus was able to spread in Western Africa so massively because the national health services and infrastructures failed. Due to a lack of information, people did not know for a long time how to protect themselves. Well-run education systems and health services, as well as functioning infrastructures, are the best protection against epidemics.

However, the path towards this ambitious goal is long and costly. A faster and more effective intervention would be the integration of epidemic prevention into all areas of development aid. In regions particularly affected by outbreaks many small and simple measures can be implemented that contribute to the protection against dangerous pathogens.

### 4.2. Early Warning System for Emerging Pathogens

Newly-emerging pathogens almost always jump from animals to humans. Ebola virus came from fruit bats or some other, yet unknown reservoir. SARS coronavirus came from palm civets, HIV-1 from chimpanzees and influenza A virus from waterfowl (While fruit bats are regarded as the most likely animal host of Ebola virus, this has not been proven yet. Severe Acute Respiratory Syndrome is a viral lung disease that claimed over 1000 victims during an international outbreak in 2003). Ebola virus may have circulated in Western Africa for many years before the actual outbreak. Equipped with simple laboratory tools and a mobile telephone, hospitals in endangered regions could act as sensors for a global surveillance system (“Global System for Early Warning and Response” (A. Kekulé, presentation at the Global Working Group, German Federal Foreign Office, 2005)). After the tsunami of 2004, an earthquake warning system was established in the Indian Ocean. The EVD outbreak in Western Africa alerts us that similar alarm bells for the global threat of pandemics are urgently needed.

### 4.3. African Center for Disease Control and Prevention

The prevention, early detection and control of outbreaks are managed by specialized centers for disease control in almost every continent. The CDC in Atlanta, which may well be seen as the most appropriate blueprint for such an organization, operates with 15,000 employees and an annual budget of US$ 6.9 billion. Other regions of the world are overseen by similar, albeit much smaller, disease control institutions, e.g., the Chinese Center for Disease Control and Prevention (CCDC) in Beijing or the European Centre for Disease Prevention and Control (ECDC) in Sweden. Australia has invested in decentralized academic centers to lead research and response in similar areas to the US CDC rather than building up a single state-owned institution.

For Africa, however, neither a common center for disease control nor a decentralized network equivalent exists. Taking into account that the rural regions of this continent are supposed to be home to many of the most dangerous pathogens on earth, an African Center for Disease Control and Prevention (“ACDC”) is urgently needed.

The idea of an African CDC was brought up already in 2013, at the African Union Special Summit on HIV, AIDS, Tuberculosis and Malaria in Abuja, Nigeria. However, it was not seriously followed thereafter, due to both financial and political restraints. However, now, in the aftermath of the Ebola outbreak, the ACDC concept has been revived.

The present idea is that an ACDC should copy the concept of the European ECDC: A small coordinating and advisory office, with its own laboratories and field intervention task forces. However, help for Africa and protection of the rest of the world does not come cheaply. In contrast to Europe, where many states have powerful national disease control facilities (such as Robert Koch Institute in Berlin, Public Health England in London and the Pasteur Institute in Paris), almost no specialized expertise exists in African countries. Therefore, an African CDC should run its own, state-of-the-art laboratories and intervention teams.

### 4.4. Medical Response Unit

The fast setup of ETCs, in sufficient numbers, is crucial for the early containment of an outbreak. In Western Africa neither WHO, the international community of states, nor single countries were able to implement this. In recognition of this shortcoming, suggestions for an international Medical Response Unit, which could be deployed quickly in case of an outbreak, were made (“Humanitarian Intervention Troup” (Alexander Kekulé, 18 September 2014); “White Coat Corps” (Ban Ki-moon, 25 September 2014); “White Helmet Troup” (Frank-Walter Steinmeier, 20 October 2014)).

The necessary technical equipment is not a challenge. Mobile units in tents or containers have proved to be superior to fixed buildings. Corresponding resources could be made available by the UN Disaster Assessment and Coordination (UNDAC), the European Commission Humanitarian Aid and Civil Protection (ECHO), the IFRC and national military and disaster aid units. The provision of consumable materials (e.g., protection suits, disinfectants) should be secured through supply contracts or stockpiling, respectively.

In addition, ETCs need access to simple lab equipment for the determination of basic parameters, in particular electrolytes (salt concentrations in blood and urine). Also plasma extraction (plasmapheresis) devices are important because antibodies from the blood of convalescent patients could potentially be used as a therapy for the newly-infected.

A far greater challenge than equipment is the personnel. For a treatment center with 100 beds, around 30 international and 300 local aid workers are required. In contrast to IFRC, MSF and other non-governmental organizations, states have no access to pools of field workers who are already trained and can be deployed quickly. As well, government-led missions involving voluntary personnel face considerable legal problems in most countries (liability in case of infection, leave from the workplace, *etc*.).

For the Medical Response Unit proposed here, four main components are necessary:

- An international operational command. It develops and practices scenario-based, generic plans for medical crises and acts as a coordinator in case of an outbreak.
- An interdisciplinary roster of experts. In case of an outbreak, consultants, trainers and field leaders can be called up from this pool.
- A network of cooperating organizations providing technical and logistical support (e.g., UNDAC, ECHO, IFRC, national military and technical aid units).
- National voluntary aid worker pools from relevant professions. These should be adequately prepared through continuous education (disaster medicine, languages, *etc*.) and, when necessary, through special field training.

The Medical Response Unit should as a rule act in conjunction with aid organizations in the affected region, which in particular provide the necessary field staff. Such public-private cooperative efforts have proven very effective during the Western African outbreak.

## 5. Rapid Outbreak Intervention Cannot Be Led by WHO

The shortcomings of WHO during the EVD crisis can only be partially blamed on particular persons. More important is a structural problem which cannot be solved in the medium term. The yearly budget of the global health guardians, which has remained unchanged for years, stagnates at a meager US$ 2 billion (as a comparison: the US health authority CDC alone has access to US$ 6.9 billion yearly). Of this budget, assessed contributions of the member states account only for 23 percent. The rest comes from voluntary donations, which usually are tied to specific projects. The dependency on voluntary donations made the agency highly susceptible to political interference. According to the demands of the industrial states, which contribute comparatively large amounts, ailments of civilization like coronary heart disease, diabetes and obesity have lately been pushed to the top of the WHO agenda. In the case of an outbreak, the concerned country and regional offices set the pace of the WHO response, in accordance with the demands of the national governments.

With the International Health Regulation*s* (IHR) of 2005, the main responsibility for epidemic control was transferred from WHO onto the member states. This made sense in the light of the SARS epidemic of 2003, which had been the reason for the 2005 revisions of the IHR, because mainly rich countries were affected. On the same grounds, the PHEIC essentially provides measures for the border protection of highly developed states (e.g., restrictions of air and maritime traffic). For the containment of outbreaks within developing countries, however, WHO has no effective policy in place.

To ensure effectiveness and speed as well as financial and political independence, the Medical Response Unit must be established outside WHO; for example, as a continuation of the UNMEER program of the United Nations or an interdisciplinary unit within the European Commission. In regard of its geographic vicinity and colonial heritage, Europe has both special security demands and special responsibilities for Africa.

The four measures proposed here could be funded by the Pandemic Emergency Facility (PEF), a novel financial instrument currently developed by the World Bank as a reaction to the EVD epidemic. In addition, the industrialized countries should guarantee, by national legislation, that voluntary aid workers are properly insured and their employers are compensated for their absence.

The world can count itself lucky that Ebola virus did not spread any further, in particular after the satellite outbreak in Nigeria. A virus only slightly more contagious could have caused a worldwide pandemic, claiming millions of victims. With the globalization of travel and goods, the war between man and microbes has exacerbated to a level that is unprecedented in history. In this “New Age of Pandemics”, the Western African EVD outbreak was just the beginning. At risk, from now on, is no less than the survival of humanity. In this war, at the outmost border of the human species, epidemic control in developing countries is our last line of defense.

